# Effects of environmental enrichment and caloric restriction on hippocampal changes in early adult rats

**DOI:** 10.7586/jkbns.24.033

**Published:** 2024-11-25

**Authors:** Joo Hee Lee, Youn-Jung Kim

**Affiliations:** 1 Korea Armed Forces Nursing Academy, Daejeon, Korea; 2 College of Nursing Science, Kyung Hee University, Seoul, Korea

**Keywords:** Environment, Caloric restriction, Hippocampus, Anxiety

## Abstract

**Purpose:**

This study used an animal model to examine the effects of environmental enrichment (EE) and caloric restriction (CR) on hippocampal changes in early adulthood in a rat model.

**Methods:**

Male Sprague-Dawley rats were randomly assigned to control, EE, and CR groups. After 8 weeks of EE and CR, behavioral, biochemical, and molecular biological assessments were performed. Behavioral tests included the open field test for anxiety-like behavior, the eight-arm radial maze test for spatial learning, and the passive avoidance test for short-term memory. Glucose tolerance was assessed with an intraperitoneal glucose tolerance test, and the molecular markers associated with neuroinflammation were evaluated.

**Results:**

Both EE and CR reduced anxiety-like behaviors, as evidenced by increased time in the central region of the open field test and decreased rearing. However, neither EE nor CR significantly improved short-term memory or spatial learning. Nonetheless, the CR group showed a decrease in eight-arm radial maze completion time, indicating potential for enhanced learning. Both interventions improved glucose tolerance, with lower fasting blood glucose levels in the CR and EE groups. Molecular biological analyses showed that neuroinflammatory markers interleukin-6 and inducible nitric oxide synthase were reduced in the EE and CR groups and that Iba-1 had anti-inflammatory effects due to its neuroprotective action.

**Conclusion:**

EE and CR were beneficial for emotional and metabolic health in early adult rats due to reductions in anxiety-like behaviors and neuroinflammation with a concomitant improvement in glucose metabolism. However, the effects of these modalities on improving cognitive function were limited, illustrating the need for further research.

## INTRODUCTION

In the past, when life expectancy was shorter, people’s health was largely determined by genetics, and it was considered natural to either be healthy or not. However, the current global trend of aging means that most people will live longer as elderly individuals, increasing vulnerability to chronic diseases such as cancer, cardiovascular diseases, Alzheimer’s disease, Parkinson’s disease, and diabetes. According to the Korea Disease Control and Prevention Agency, 7 out of the 10 leading causes of death in Korea are chronic diseases, and elderly individuals aged 65 and older on average suffer from two or more chronic diseases [1,2]. Chronic diseases typically appear in midlife after long-term exposure to smoking, alcohol, stress, physical inactivity, high-fat diets, and unhealthy lifestyles. Alzheimer's disease, the most common form of dementia, is a chronic neurodegenerative disease that begins with brain damage long before cognitive symptoms appear, accumulating over time [3]. It is known that hypertension, diabetes, and cerebrovascular diseases increase vulnerability to this condition. [4].

Early adulthood is the period of human life between the ages of 20 and 40 that marks the peak of physical and psychosocial development and is associated with many events that lead to the experience of health risk behaviors, including binge drinking, poor sleeping habits, smoking, and substance use [5]. The potential consequences of health risk behaviors in early adulthood can lead to stratification of chronic disease in later life. An analysis of the National Longitudinal Study of Adolescent to Adult Health in the United States found that a healthy lifestyle in young adulthood is significantly associated with cardiovascular risk. Even though harmful behaviors may show resilience in the early years, they have lasting physical and mental impacts over time [6]. Health behaviors are not isolated phenomena but consist of routines and habits that comprise a lifestyle. Physical activity and regular exercise have been shown to positively impact risk factors for chronic diseases such as cardiovascular disease, type 2 diabetes, obesity, and cancer [7-10].

Environmental enrichment (EE) is an experimental paradigm designed for rodents, characterized by increased sensory, cognitive, and physical stimulation. Animals in EE environments are exposed to conditions that promote physical, social, and cognitive development [11]. The application of EE has been shown to enhance neurogenesis, synaptic plasticity, and overall hippocampal function [12,13]. Previous studies have highlighted the benefits of EE on hippocampal volume, synaptic density, and expression of neurotrophic factors in rodents, reporting potential protective effects against age-related cognitive decline, or the strength of EE applied early in life (2-21 days of age) [14-16]. However, further research is needed on the effects of EE initiated in early adulthood in order to translate and apply findings from animal studies to humans. Another potential modulator of brain health, calorie restriction (CR), has been widely studied for its role in extending lifespan and delaying the onset of age-related diseases. CR has also been found to be associated with neuroprotective effects, including improved insulin sensitivity, reduced oxidative stress, and enhanced autophagy [17,18]. Animal studies have shown that CR can affect neuroplasticity and cognitive performance in the hippocampus [19], but the specific consequences of CR in early adulthood are not yet fully understood, as most studies have been conducted in the context of aging.

Neuroinflammation is a coordinated response of the central nervous system (CNS) to harmful stimuli or damage, which occurs in various neurodegenerative diseases and infections. Microglia and astrocytes are key regulators of CNS inflammatory responses, releasing inflammatory mediators [20]. Under normal conditions, microglia have a distinctive branched morphology with small, thin projections extending from the cell body into the surrounding environment. Upon activation in response to brain injury or immune stimulation, microglia undergo dramatic morphological changes, with excessive activation exacerbating inflammatory responses by increasing pro-inflammatory cytokine levels [21,22]. Astrocytes, characterized by the expression of the intermediate filament protein glial fibrillary acidic protein (GFAP), stabilize the cytoskeleton, and changes in GFAP expression can lead to abnormal synaptic plasticity [23]. CNS inflammation may also be induced or exacerbated by peripheral factors such as systemic inflammatory mediators, insulin resistance, and other metabolic conditions, which activate microglia and astrocytes [24]. Therefore, factors such as physical activity, environment, and diet can play a critical role in modulating CNS inflammation.

Early adulthood represents a crucial period for health behavior interventions, and basic research exploring the effects of lifestyle factors on health during this time is important not only for its own sake but also for understanding future health risks. This study aims to deepen the understanding of health interventions by examining the effects of environmental and dietary changes on molecular biological changes in the brain through animal experiments. We hypothesized that applying EE and CR to early adult rats would positively affect cognitive function and emotional responses related to anxiety by maintaining appropriate blood glucose levels and reducing neuroinflammation in the hippocampus. We hypothesized that the application of EE and CR to early adult Sprague-Dawley (SD) rats would have a positive effect on cognitive function and anxiety-related emotional responses by maintaining adequate blood glucose levels and reducing neuroinflammation in the hippocampus.

## METHODS

### 1. Study design

**Figure 1.**
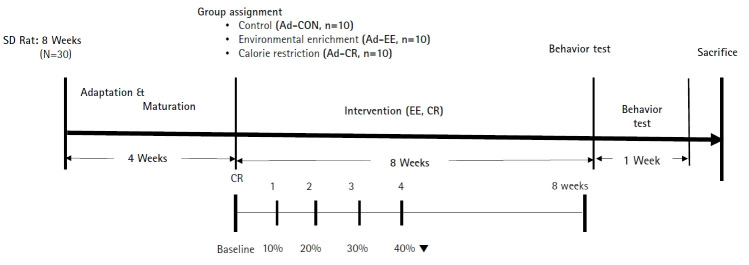
Experimental design. EE = Environmental enrichment; CR = Calorie restriction; Ad-CON = Adult control; Ad-EE = Adult EE; Ad-CR = Adult CR; SD = Sprague-Dawley.

This is a randomized control group post-test only design, purely experimental study to determine the effects of environmental determinants EE and CR on health. The groups are Adult Control (Ad-CON), Adult EE (Ad-EE), and Adult CR (Ad-CR). Rats are typically considered adults at 8 weeks of age, and it has been reported that SD rats begin to show signs of aging by 7 months of age due to decreased skeletal growth [25]. Therefore, in this study, 8-week-old rats were housed and allowed to mature and adapt to the environment for 4 weeks before being randomized into groups at 12 weeks of age. The EE and CR interventions were then applied for 8 weeks, followed by 1 week of behavioral testing. The EE and CR interventions were maintained for 1 week during the behavioral test, and an intraperitoneal glucose tolerance test was performed after the behavioral test. The animals were then allowed to rest and sacrificed the next day to collect blood and tissue for molecular biology experiments (Figure 1).

### 2. Animals

Eight-week-old male SD rats were obtained from Orient Bio. According to the calculation of Mead's resource equation for selecting the number of animals, E = total number of animals-total number of groups, and the value of E is to be determined between 10 and 20 with a degree of freedom of error [26]. Since this study has three groups, five to eight animals are required for each group. However, considering the processing for post-experimental protein analysis and immunohistochemistry, 10 animals were assigned to each group. The animal housing was maintained at a constant temperature (25 ± 2℃), humidity (60 ± 5%), and light for 12 hours per day according to the circadian rhythm. The animal cages were provided with clean bedding that was changed once a week by the same person to create a comfortable environment, and two animals were housed per cage.

### 3. EE

EE for rodents is defined by several criteria, including high cages and gnawing objects, shelters and nests, and social housing to provide sufficient space for the animals to be active [27]. The EE applied in this study was a large cage (80 × 50 × 100 cm) in which five animals were housed for socialization and provided with toys, wooden blocks, balls, running wheels, and hiding places. These objects were changed weekly to provide different stimuli.

### 4. CR

Since there is no recommended caloric intake for experimental animals, the amount of food consumed by each individual was measured 1 week before group assignment and averaged. The Ad-CON and Ad-EE groups were allowed to eat ad libitum, but the Ad-CR group was gradually restricted by 10% in the first week, 20% in the next week, 30% in the following week, and finally 40%, which was maintained until the end of the experiment.

### 5. Behavior test

#### (1) Open-field test (OFT)

The OFT is used to analyze locomotor activity and anxiety levels in rodents. The testing environment was set to a slightly dim temperature of 23°C to minimize environmental influences, and animals were moved to the testing room 1 hour before the test to allow for adaptation. The open-field box (90×90×50 cm) had a grid pattern on the floor, dividing it into 16 squares, with the central 4 squares considered the center region. The animals were placed in the open field box, allowed 60 seconds to adapt, and their behavior was observed for 5 minutes. Movements in the side and central regions were recorded. After the test, animals were returned to their cages, and the open field box was cleaned with 70% ethanol to remove odors and prevent cross-contamination.

#### (2) Eight-arm radial maze test

The eight-arm radial arm maze (RAM) test consists of a central platform with eight arms radiating outward. The walls of the maze are made of transparent material, allowing the rats to see visual cues, such as desks, walls, and pictures in the testing room. A small amount of food was placed at the end of each arm to motivate the animals to explore. At the start of the experiment on day 1, the animals are placed in the center of the maze and given 15 minutes to explore the perimeter of the maze. On the first and second days, food was placed at the ends of all arms to allow the animals to familiarize themselves with the maze. On the third day, the food was removed, and the final test was conducted. The time taken to explore each arm and the number of correct (arms visited without repeating) were recorded, with a maximum of 8 minutes allowed for each trial. The maze was cleaned with 70% ethanol after each test to remove odors.

#### (3) Passive avoidance test (PAT)

PAT is used to evaluate short-term memory by applying an aversive stimulus (electric shock). The equipment used was an automated dark-light box (LE870, Harvard Apparatus, Barcelona, Spain). The passive avoidance box is divided into a bright light aversive chamber that rodents dislike and a dark avoidance chamber where electric shocks are delivered from the floor, with a door that automatically closes between the two chambers. On the first day, the animals were placed in the apparatus and allowed to explore both chambers freely. When the animal entered the dark chamber, the door closed automatically, and a 0.5 mA electric shock was delivered for 3 seconds. The next day, the test was repeated without the electric shock, and the time it took for the animal to move from the light to the dark chamber was recorded, with a maximum latency of 300 seconds. A longer latency time was considered indicative of better short-term memory.

### 6. Intraperitoneal glucose tolerance test (IPGTT)

The IPGTT was used to assess glucose metabolism. After a 12-hour fast, baseline blood glucose levels were measured, and a 50% dextrose (JW Pharmaceutical, Seoul, Korea) solution (2 g/kg) was administered intraperitoneally. Blood glucose levels were measured at 15, 30, 60, 90, and 120 minutes post-injection using a glucose meter (Accu-Check, Roche Co., Seoul, Korea). The area under the curve (AUC) was calculated using the trapezoidal rule [28] to assess glucose tolerance.

*AUC = Σ((BST^†^))i + (BST)i-1 × ((time)i-(time)i-1 / 2)*

^†^BST = blood sugar test.

### 7. Molecular biological tests

#### (1) Brain preparation

After general anesthesia using isoflurane, the chest cavity was opened, and the heart was perfused with 0.05 *M* phosphate-buffered saline (PBS) to remove blood. The brain was then extracted, separated by regions, and stored at -70°C for further analysis. For immunohistochemistry, brains were perfused with 4% paraformaldehyde (PFA) for 10 minutes to fix the tissue, then post-fixed in 4% PFA for 24 hours at 4°C. The brains were then dehydrated in a 30% sucrose solution for 2-5 days, and frozen of 40μm sections were prepared using a cryostat (Leica, Germany).

#### (2) Western blot

Western blot was performed to quantify protein expression levels. Brain tissues were homogenized in a cold lysis buffer, centrifuged at 13,000 RPM for 20 minutes at 4℃, and the supernatant was collected. Protein concentrations were determined using the Lowry method. Proteins were separated via sodium dodecyl sulfate-polyacrylamide gel electrophoresis and transferred to a polyvinylidene fluoride membrane. The membrane was blocked with 5% skim milk and incubated overnight with primary antibodies against interleukin-6 (IL-6) (1:5000, ab9324, Abcam, Cambridge, UK) and inducible nitric oxide synthase (iNOS) (1:2500, sc-651, Santa Cruz Biotechnology, Dallas TX, USA), followed by incubation with secondary antibodies conjugated with horseradish peroxidase. Detection was performed using enhanced chemiluminescence, and bands were visualized using an imaging system. Protein levels were quantified using ImageJ software (U.S. National Institutes of Health, Maryland, USA), normalized to β-actin.

#### (3) Immunohistochemistry

Immunohistochemistry is an experiment for the diagnosis of disease or biological research by identifying the presence of specific antigens in tissues or cells through specific antigen-antibody reactions Stored hippocampal tissue sections were washed three times for 5 minutes each in 0.05 *M* PBS. Endogenous peroxidase activity was blocked by treating the sections with 3% hydrogen peroxide for 30 minutes. Afterward, sections were blocked for 2 hours to prevent non-specific binding. The primary antibodies used were anti-NeuN (1:2000, ab134014, Abcam, Cambridge, UK) to label neurons, anti-GFAP (1:1000, ab53554, Abcam, Cambridge, UK) for astrocytes, and anti- Ionized calcium-binding adapter molecule 1 (Iba-1) (1:500, ab5079, Abcam, Cambridge, UK) for microglia. Sections were incubated with the primary antibodies overnight at 4°C. After washing three times with PBS, sections were incubated with biotinylated secondary antibodies for 1 hour at room temperature. After reacting with Avidin-biotin complex (Vectastain-Elite ABC kit, Vector Laboratories, Burlingame CA, USA), Diaminobenzidine (DAB, Vector Laboratories, Burlingame CA, USA) for color development. The stained sections were then mounted onto slides, free floating, and dehydrated through a graded ethanol series (70%, 80%, 90%, and 100%) before being cleared with xylene. Finally, the slides were cover-slipped and examined under a light microscope (Olympus, Tokyo, Japan) at 100x and 400x magnification to quantify the labeled cells and assess the changes in immune markers.

### 8. Statistical analysis

All experimental results obtained in this study were repeated in triplicate and averaged, and data were expressed as the mean and standard error of the mean. Statistical analysis was performed using the SPSS 25 program (IBM Corp., Armonk, NY, USA), and normality was evaluated by the Shapiro-Wilk test, and differences between groups were tested by one-way ANOVA. Post hoc analysis was performed by Scheffe’s' test, and statistical significance was adopted at *p*-value < .05.

### 9. Ethical considerations

The policies of the Institutional Animal Care and Use Committee and the Guide for the Use of Animals of the National Institutes of Health were followed and animal experiments were conducted following the 3Rs (replacement, refinement, and reduction) principle. All participants in this study were properly trained in animal handling, injection and blood collection, and anesthetic administration, and all experimental procedures were approved by the Kyung Hee University Institutional Animal Care and Use Committee (KHSASP-21-570).

## RESULTS

### 1. Changes in anxiety-like behavior and spatial working memory as determined by behavioral testing

**Figure 2.**
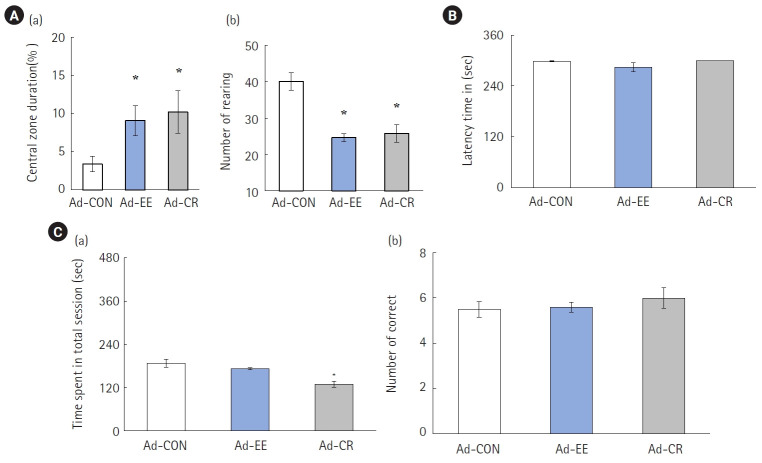
Result of anxiety-like and memory behavior test. (A-a) Total duration (%) of central zone entries of the open-field test (A-b) Number of rearing (B) Latency time (sec) to the dark box in passive avoidance test (C-a) Total time spent in eight-arm radial maze test for the session (C-b) Result of spatial working memory function in eight-arm radial maze test. Data are expressed as mean±S.E.M. S.E.M: Standard error of the mean *significantly different from Ad-CON. Ad-CON = Adult control; Ad-EE = Adult environmental enrichment; Ad-CR = Adult calorie restriction.

In an OFT performed to determine changes in the emotional responses of the rats, there was a difference between groups in the frequency with which each animal visited and stayed in the centralized area (F = 17.60, *p* < .001). When the frequency of visits to the center was calculated from the total frequency of activity, the Ad-CON group was 3.38 ± 1.01, the Ad-EE group was 9.06 ± 1.95, and the CR group was 10.19 ± 2.80%, indicating that the Ad-EE and Ad-CR groups stayed in the center more than three times as often as the control group. In addition, the number of attempts to stand on the hind legs (rearing) was significantly reduced in the Ad-EE and Ad-CR groups, from 40.10 ± 2.39 in the Ad-CON group, 24.62 ± 1.07 in the Ad-EE group, and 25.80 ± 2.41 in the Ad-CR group (F = 13.47, *p* < .001) (Figure 2-A).

Memory was assessed with two behavioral tests: the PAT assessed short-term memory and the RAM assessed spatial learning and memory. Latency time on the PAT was 298.30 ± 1.70 seconds for the Ad-CON group, 284.30 ± 10.96 seconds for the Ad-EE group, and 300 ± 0.00 seconds for the Ad-CR group, with no significant difference between groups (F = 1.84, *p* = .170) (Figure 2-B). Time spent in RAM was 187.50 ± 11.33 seconds for the Ad-CON group, 171.89 ± 3.24 seconds for the Ad-EE group, and 128.26 ± 8.36 seconds for the Ad-CR group, with the Ad-CR group completing the test the fastest (F = 13.55, *p* < .001), and there was no statistical difference between the Ad-EE group and the Ad-CON group. The number of correct was 5.50 ± 0.34 for the Ad-CON group, 5.60 ± 0.22 for the Ad-EE group, and 6.00 ± 0.45 for the Ad-CR group, with no difference between groups (F = 0.57, *p* = .057) (Figure 2-C). Thus, Ad-EE and Ad-CR reduced anxiety-like behavior in rats and did not affect spatial learning and working memory.

### 2. Changes in blood glucose concentration

**Figure 3.**
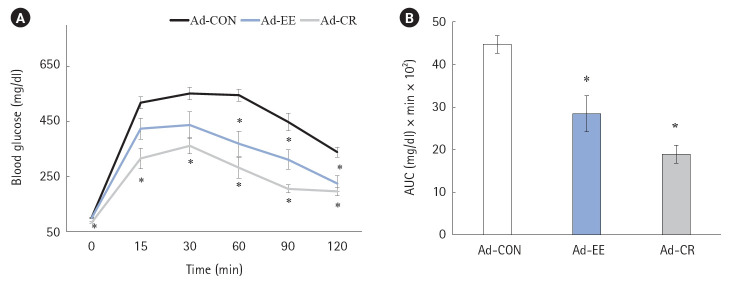
Result of intra peritoneal glucose tolerance test (IPGTT). (A) Variations in glucose levels during IPGTT (B) Area under curve (AUC) for IPGTT. Data are expressed as mean ± S.E.M. S.E.M: Standard error of the mean *significantly different from Ad-CON. Ad-CON = Adult control; Ad-EE = Adult environmental enrichment; Ad-CR = Adult calorie restriction.

To assess the effects of Ad-EE and Ad-CR on blood glucose levels, animals were fasted for over 12 hours, and changes in blood glucose were measured over time. Fasting blood glucose levels were significantly lower in the Ad-CR group (82.20 mg/dL) compared to the Ad-CON group (100.00 mg/dL) and the Ad-EE group (98.40 mg/dL) (F = 18.60, *p* < .001). In the IPGTT, the Ad-CR group showed significant differences in blood glucose levels at all time points compared to the Ad-CON group, while the Ad-EE group showed significant differences from the 90-minute mark onward (Figure 3-A). The AUC calculated from the IPGTT results showed a significant decrease in the Ad-EE and Ad-CR groups compared to the Ad-CON group (F = 19.31, *p* < .001), although there were no significant differences between the Ad-EE and Ad-CR groups (Figure 3-B).

### 3. Molecular changes in the hippocampus

**Figure 4.**
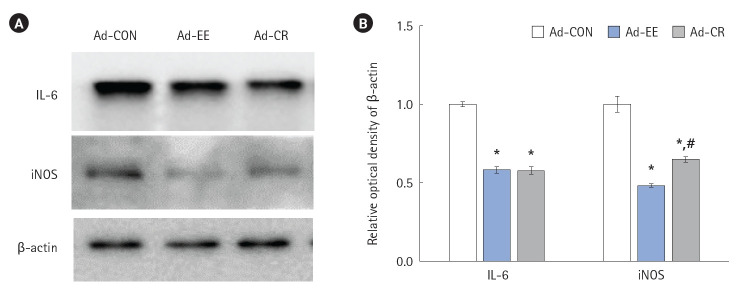
Effects of EE and CR on IL-6, iNOS immunoreactivity of hippocampus. (A) Western blot analysis of IL-6, iNOS (B) Relative optical density of IL-6, iNOS. Data are expressed as mean ± S.E.M. S.E.M: Standard error of the mean *: significantly different from Ad-CON, #: significantly different from Ad-EE. Ad-CON = Adult control; Ad-EE = Adult environmental enrichment; Ad-CR = Adult calorie restriction; IL-6 = Interleukin-6; iNOS = Inducible nitric oxide synthase.

To investigate molecular changes related to inflammation in the hippocampus, the expression of IL-6 and iNOS was assessed using western blot and normalized to β-actin. (Figure 4-A). IL-6 was reduced by half in the Ad-EE and Ad-CR groups compared to Ad-CON expression, which was statistically significant (F = 48.76, *p* < .001), with no significant differences between these groups. For iNOS, the expression was reduced by less than half in the Ad-EE group compared to the Ad-CON group (F = 137.63, *p* < .001), and there was also a significant difference between the Ad-CR and Ad-EE groups, with the greatest reduction in the Ad-EE group (*p* = .043) (Figure 4-B).

**Figure 5.**
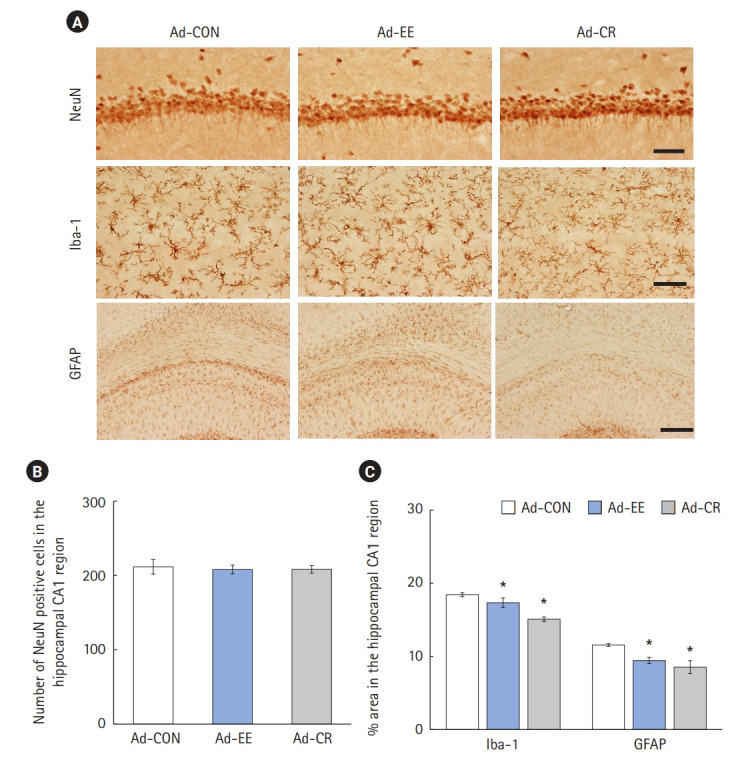
Effects of EE and CR on immunoreactivity of hippocampus CA1 region. (A) Photomicrographs of NeuN, Iba-1, GFAP on the hippocampus CA1 region. (B) The number of NeuN positive cells on CA1 region. (C) The % area of Iba-1, GFAP positive cells on CA1 region. Data are expressed as mean±S.E.M. S.E.M: Standard error of the mean *significantly different from Ad-CON. Ad-CON = Adult control; Ad-EE = Adult environmental enrichment; Ad-CR = Adult calorie restriction; NeuN = Neuronal nuclear antigen; Iba-1 = Ionized calcium-binding adapter molecule 1; GFAP = Glial fibrillary acidic protein. (Scale bar = 20 μm).

The histological analysis of the CA1 region of the hippocampus, a key area for learning and memory, showed no significant differences in the number of neurons between the experimental groups (F = 0.08, *p* = .929). The Ad-CON group had an average of 211.67 ± 9.76 neurons, the Ad-EE group 208.00 ± 5.86, and the Ad-CR group 208.17 ± 6.66 (Figure 5-A, 5-B). The % area of positive immunoreactivity for Iba-1, a marker of microglia, was 18.45 ± 0.26 in the Ad-CON group, 17.34 ± 0.18 in the Ad-EE group, and 15.09 ± 0.30 in the Ad-CR group, all significantly reduced in the experimental groups (F = 45.62, *p* < .001). The area of positive immunoreactivity for GFAP, a marker of astrocytes, was 11.56 ± 0.65 in the Ad-CON group, 9.44 ± 0.43 in the Ad-EE group, and 8.88 ± 0.53 in the Ad-CR group, which were all significantly reduced in Ad-EE and Ad-CR compared to the Ad-CON group (F = 6.77, *p* = .008) (Figure 5-A, 5-C).

## DISCUSSION

This study aimed to investigate the effects of EE and CR on hippocampal changes in early adult male rats. The results showed that both EE and CR effectively reduced anxiety-like behavior but had limited effects on improving memory. Additionally, both interventions were found to improve glucose tolerance and reduce neuroinflammatory markers in the hippocampus.

The OFT is a well-established tool for assessing locomotor activity and emotional responses such as anxiety and was developed in 1934 to measure emotion in rodents [29]. In this test, animals tend to spend more time in the side zones when anxious, avoiding the open center. Administration of anxiolytics increases the time spent in the open areas [30]. A study by Levay et al., [31] showed that applying CR (25% and 50%) for 3 weeks in Wistar rats resulted in increased entries into the center zone of the OFT, supporting the idea that CR has anxiolytic effects. In our study, we applied a 40% CR for 8 weeks, which is consistent with previous research in terms of showing an anxiety-reducing effect. The rearing behavior (attempts to stand on hind legs), often observed during the OFT, is considered an exploratory behavior and is also used as an anxiety measure [32]. Previous studies, like Rojas-Carvajal et al., [33] found that rats subjected to 60 days of EE showed a reduction in rearing behavior compared to those housed in standard cages, suggesting that repeated exposure to the OFT reduces rearing, which supports the findings of the present study that EE reduces anxiety-like behavior.

In humans, the cerebral cortex processes information received from the environment and interacts with the hippocampus to encode new information and the amygdala to encode emotional aspects of cognitive processing [34]. The hippocampus's role in both human and rodent brains is similar in that it affects spatial memory and emotional responses within the limbic system [35]. This study used the PAT and RAM to assess the effects of EE and CR on learning and memory. In the PAT, which measures short-term memory, no significant differences were observed between the groups, and in the RAM, only the Ad-CR group showed a reduction in the total time taken to complete the test. To assess working memory in the RAM, animals are required to navigate each maze only once, and navigating the same maze more than once can be seen as evidence of working memory impairment [36]. However, in this study, the number of correct responses in the RAM did not differ between groups, indicating that EE and CR applied in early adulthood did not significantly improve memory at this time. This is in contrast to a study by Hullinger et al., [37], who reported that EE applied to 2-month-old F344 rats for 4 months improved learning and memory in the Novel object recognition test and Morris water maze test. However, the differences in results may be attributed to variations in the rat strains used, the age at which EE was applied, and the type and duration of memory tests. Additionally, in some studies, such as by Hullinger et al., [37] EE was applied over a longer period, which may have contributed to the observed cognitive improvements. CR, defined as reducing calorie intake by 20%~40% without inducing malnutrition, has been shown to promote metabolic fitness and longevity, particularly in aging rodent models [38]. It has also been linked to improvements in learning and memory. However, previous research suggests that CR does not necessarily promote neurogenesis in younger animals [39], which aligns with the findings of this study. For example, mild (30%) and intense (60%) CR applied to Wistar rats for 15 weeks enhanced learning performance in the Y-maze but limited improvement in memory [40]. Additionally, another study found that applying a 15% CR starting from postnatal day 28 increased brain-derived neurotrophic factor levels and improved spatial learning in female rats [41], but these results differed depending on species, strain, and the method and duration of dietary restriction. Taken together, the findings of this study suggest that applying EE and CR during early adulthood, a period when memory deficits may not yet be evident, suggests that the effect on memory improvement is difficult to compare.

The effects of EE and CR on metabolic health were evaluated through changes in blood glucose levels. Recent trends in CR research have focused on its effects in disease models, such as a study where 30% CR applied to diabetic animal models for 20 weeks improved insulin resistance and protected pancreatic function [42]. The significance of this study is that the effect was observed in a non-disease state and the duration of the intervention was relatively short. In this study, we observed that both EE and CR interventions improved glucose tolerance, with a significant reduction in the AUC for both groups during IPGTT, suggesting improved glucose metabolism. In the EE group, fasting blood glucose did not differ from the control group, but glycemia levels improved after 90 minutes on the IPGTT, similar to the effect of direct CR. A recent study reported that EE application to adolescent-aged 4-week-old mice for 14 weeks enhanced glucose-induced feeding inhibition by enhancing glial cell line-derived neurotrophic factor gene expression in the hypothalamus [43]. In addition, long-term EE application to middle-aged mice reduced glucose tolerance, body fat, and increased muscle mass [44], suggesting that the beneficial effects of EE span a range of ages. The difference in IPGTT results between the Ad-EE and Ad-CR groups can be attributed to the distinct physiological effects of each intervention. The Ad-CR group had a lower mean body weight due to CR, which typically enhances insulin sensitivity and glucose tolerance (data not shown), supported by evidence that fat loss improves glycemic control [45]. Conversely, the Ad-EE group likely had increased energy expenditure from enhanced physical activity, raising their metabolic rate and resulting in higher glucose tolerance levels. Exercise has been shown to enhance mitochondrial function and energy metabolism, improving glucose utilization even without significant weight loss [46]. Thus, the differences in glucose tolerance observed in this study may be due to weight reduction in Ad-CR and increased energy expenditure in Ad-EE. Further detailed measurements of metabolic parameters would provide a clearer understanding of these effects. These findings indicate that EE and CR in early adulthood can positively influence glucose metabolism, potentially reducing the risk of metabolic-related functional decline after early adulthood.

Molecular analysis of the hippocampus showed that inflammatory markers such as IL-6 and iNOS were significantly reduced in both the Ad-EE and Ad-CR groups. IL-6 expression was approximately half that of Ad-CON in both intervention groups, while iNOS was significantly reduced in the Ad-EE group, suggesting a potent anti-inflammatory effect of Ad-EE. Immunohistochemical analysis of microglia and astrocyte markers (Iba-1 and GFAP) showed a decrease in both Ad-EE and Ad-CR groups. Microglial activation can be divided into M1 classical phenotype and M2 alternative phenotype. M1-type microglia are activated by the expression of molecules including CD16 and CD32, while M2-type activation produces anti-inflammatory cytokines (IL-10, TGF-β) and various growth factors (IGF-1, FGF, NGF, GDNF) to promote phagocytosis and support neuronal survival. The decreased effect of Iba-1 on CR and EE in this study can be attributed to the cytoprotective action of microglia. Recent studies have highlighted the interactive roles of microglia and astrocytes during inflammation. One study on subarachnoid hemorrhage found that Iba-1-positive microglial activation is influenced by GFAP-positive astrocytes, suggesting that astrocytes play a regulatory role in modulating microglial activity during neuroinflammation [47]. Our findings are consistent with this, as both EE and CR led to decreased expression of Iba-1 and GFAP, indicating reduced activation of these glial cells These results emphasize the capacity of interventions like EE and CR to modulate both microglial and astrocytic activation, thereby reducing neuroinflammation and contributing to improved brain health. Considerable research has been conducted on the role of EE in enhancing neuroplasticity. A recent review demonstrated that EE promotes neuroplasticity by inducing morphological and cellular adaptations, such as increased dendritic complexity and improved synaptic function, which are beneficial not only for normal brain development but also for neuronal injury recovery [48]. The anti-inflammatory effects of EE create an environment conducive to neuroplasticity, as decreased inflammatory markers like iNOS and Iba-1 enable the brain to better adapt and recover. This interaction between reduced neuroinflammation and enhanced neuroplasticity highlights the role of EE in fostering a resilient neural environment. Similarly, Kimura et al. [49] showed that EE can attenuate neuroinflammation by shifting microglial activation from a pro-inflammatory (M1) to an anti-inflammatory (M2) state. This shift reduces levels of inflammatory mediators such as TNF-α and IL-6, thereby promoting a neuroprotective environment that supports increased neurogenesis and synaptic plasticity—processes essential for learning, memory, and overall cognitive function. Additionally, EE was found to enhance dendritic branching and synaptic density in the hippocampus, a region critical for cognitive and emotional regulation. Our findings align with these studies, showing that both EE and CR were associated with reductions in inflammatory markers, including IL-6, iNOS, Iba-1, and GFAP, indicating decreased activation of both microglia and astrocytes. By reducing glial activation, EE fosters an anti-inflammatory environment that supports neuroplastic changes, ultimately promoting better cognitive outcomes and enhancing mental resilience. These findings suggest that lifestyle interventions focused on EE, such as increasing opportunities for physical activity, social interaction, and cognitive engagement, may serve as effective strategies to modulate neuroinflammation without relying solely on CR.

Despite the biological similarities, there are limitations to directly applying results from rodent experiments to humans. While EE is an experimental paradigm designed to replicate positive life experiences, it is difficult to control humans as well as in a laboratory setting, and the appropriate degree and duration of CR remains controversial, especially in young adults. However, behavioral, physiological, and molecular biological studies using animals are invaluable as a foundation for disease research, and the results of this study provide a foundation for further research into the potential of lifestyle interventions to promote cognitive-related brain health and resilience. More research is needed to determine whether these findings are directly applicable to humans and how such interventions could be practically and safely applied. Additionally, the molecular mechanisms underlying the anti-inflammatory effects of EE and CR, particularly their neuroprotective effects in early adulthood, remain unclear and warrant further investigation.

## CONCLUSION

In conclusion, both EE and CR interventions applied in early adulthood demonstrate significant potential for enhancing anti-inflammatory responses, improving glucose metabolism, and triggering neuroprotective mechanisms, which ultimately reduce anxiety-like behaviors. This highlights the neuroprotective and anti-anxiety benefits of EE and CR when implemented during early adulthood, providing a valuable insight into their potential applications as non-pharmacological approaches to promote brain health and resilience.

## Data Availability

Please contact the corresponding author for data availability.
